# Immunisation decision-making and barriers to vaccine uptake among children under-5 in limited-resource settings

**DOI:** 10.1038/s41598-025-33121-4

**Published:** 2025-12-19

**Authors:** Gbadebo Collins Adeyanju

**Affiliations:** 1 Media and Communication Science, University of Erfurt, Erfurt, Germany; 2 Centre for Empirical Research in Economics and Behavioural Science (CEREB), University of Erfurt, Erfurt, Germany; 3 German Alliance for Global Health Research (GLOHRA), Berlin, Germany; 4 Psychology and Infectious Diseases Lab (PIDI), University of Erfurt, Erfurt, Germany; 5 Adjunct Professor of Global Health, RHIBMS University, Buea, Cameroun

**Keywords:** Nigeria, Parents, Children, Immunisation, Low-resource setting, Sub-Saharan africa, Masculinity, Gender, Behaviour, Misinformation, Diseases, Health care, Risk factors

## Abstract

Immunisation coverage remains a fundamental challenge in sub-Saharan Africa, which still has the highest under-five mortality rate in the world, due to wide-ranging drivers that have complicated interventions. Unless the knowledge gaps affecting childhood vaccination decision-making are identified and effectively addressed, low immunisation coverage will persist in the region. This study aims to assess the factors influencing immunisation decision-making among caregivers of children Under-5 years old, and to understand the behaviours that shape these influences. The study employed qualitative methods, such as focus group discussions. Participants were caregivers of children Under-5 years old in Nigeria. A simplified cluster sampling approach was used to select caregivers from four geographical clusters. A minimum of seven caregivers from each cluster were purposively included. The data were analysed deductively using a meta-aggregation approach. The findings show that caregivers’ immunisation decision-making are mainly motivated by: inadequate knowledge about childhood immunisation, especially the conflict between vaccine-preventable and non-vaccine-preventable diseases; masculinity (attitudes of fathers or men can help or hinder immunisation); the child’s gender (the perception of stronger versus weaker sex); misinformation about immunisation (especially the perception that it is a form of family planning). Other influences include the exploitation of caregivers by healthcare workers; incessant stock-outs of vaccines leading to complacent behaviour associated with vaccine hesitancy; religious beliefs; poor attitudes of healthcare workers; among other factors. The factors influencing immunisation decision-making in limited-resource settings and the motivations shaping these behaviours are largely psychological, socio-cultural, behavioural, health system and structural. Interventions designed to address the root causes of gender inequity must start with men’s attitudes and the socio-cultural practices that enable them. Furthermore, the sandwich model for addressing vaccine misinformation seems promising in countering myths and conspiracies about vaccines.

## Introduction

Since the turn of the millennium, there has been unprecedented growth in childhood vaccination coverage against preventable infectious diseases in sub-Sahara Africa (SSA)^1^. Since their introduction into the National Immunisation Programme (NIP) in the 1970s, vaccines have saved over 50 million lives in the SSA region^2^. For every infant life saved during this period, about 60 years of life have been added^2^. However, despite these remarkable public health breakthroughs, acceptance and uptake still have a steep height to climb in the region, as it accounts for the highest under-five (Under-5) mortality rate globally, and accounting for 40% of total deaths in this age group^3^. Vaccination coverage in SSA has stagnated at pre-pandemic levels and, in some cases, has declined^4–6^. About one in every five children in SSA have not received basic vaccines, resulting in over 30 million children Under-5 years suffering from vaccine-preventable diseases (VPDs) every year in the region, and out of these, over half a million die annually, thereby constituting about 58% of global VPD-related deaths^7^. Furthermore, over 80% of the global zero-dose children are in the SSA, with the top ten contributing countries being Nigeria (30.1%), Ethiopia (14.1%), the Democratic Republic of the Congo (9.5%), Angola (7.2%), the United Republic of Tanzania (3.6%), Madagascar (3.4%), Mozambique (3.6%), Mali (2.4%), Chad (2.9%) and Cameroon (3.1%)^7^.

The coverage of childhood immunisation in Nigeria varies across states and regions, but generally shows the need for greater efforts, as immunisation coverage remains below the target, thereby putting a significant number of children at risk of an outbreak of VPDs [8.9]. Nigeria is among the countries in SSA with third dose Diphtheria, Pertussis and Tetanus (DPT3) coverage below 80%, and has the highest number of children not vaccinated for DTP1 (zero-dose); over 30% of these children are from Nigeria alone^7–9^.

Several factors influence childhood immunisation decision-making in Nigeria. For example, age, region, level of education, wealth index and the number of antenatal care (ANC) clinic visits are major determinants of completing the childhood immunisation schedule^5^. Other factors associated with the number of childhood vaccine doses taken include maternal age, education, income status, ANC attendance, employment, use of skilled birth attendants, religion, poverty, literacy and more^8^. The immunisation behaviour of caregivers is also influenced by social factors such as spousal opposition, institutional factors such as problems with the health system and cognitive factors such as concerns about vaccine safety^10^. Psychological antecedence such as confidence, complacency, constraints, calculation, collective responsibility, religious beliefs, rumours and more shape the behaviour of caregivers with regard to childhood immunisation^11–14^. Other factors noted at the family and community levels that contribute to low demand for immunisation include a lack of understanding of its value, perceptions of immunisation, inadequate cold chain equipment in public healthcare facilities, political crises affecting communities, vaccine availability, and fear, among others^15–18^.

Several studies in SSA, have linked the phenomenon of low vaccine uptake to misinformation, gender disparities, and masculinity norms, among other factors^12–14,19–23^. However, there is dearth of empirical, in-depth research on these factors, particularly with regards to qualitative exploration of fathers, gender duality and misinformation. There is also insufficient understanding of how these factors influence immunisation decision-making in households in limited-resource settings, such as Nigeria. Recent studies have shown that caregivers’ intention to vaccinate newborns decreases when they believe rumours or misinformation, especially that vaccines can cause infertility or are designed to reduce the population^14,18,19,21^. In addition, the opinions and actions of male partners or fathers are crucial in the overall household decision-making process, particularly in the SSA, where some studies have cited a lack of enthusiasm for vaccinating children among them^3,12,19^. Their knowledge, attitude and/or beliefs shape the household’s decision on whether or not to vaccinate a child. Disparities in immunisation coverage between boys and girls also demonstrate how the gender of children can influence vaccination decision-making^24–26^. This has also been observed outside of SSA, e.g., in Bangladesh, where girls are less likely to be fully vaccinated than boys^26^.

Even where successes have been recorded in terms of immunisation coverage and substantial uptake, progress made in the last decade is being eroded in SSA and Nigeria owing to vaccine hesitancy and other wide-ranging drivers, as well as other unclassified determinants^13,14,19,20,22,27–29^. Unless the scientific evidence needed to design effective interventions to address childhood vaccination decision-making is empirically studied, identified, measured and addressed, low vaccination demand and disproportionate infant mortality will continue to plague the region.

As mentioned earlier, the reasons for low immunisation coverage among caregivers are often complex, multifaceted and context-specific. They generally include social, institutional, psychological and cognitive factors, some of which underscores vaccine hesitancy^10^. Undoubtedly, vaccine hesitancy continues to be a significant factor affecting vaccination uptake and coverage in Nigeria^12,19,28^. A lack of trust in public institutions (16%) and the prevalence of vaccine conspiracy theories, mostly relating to religion and biotechnology (46%) has been identified as triggers associated with vaccine hesitancy in Nigeria, particularly following the introduction of the SARS-CoV-2 (COVID-19) vaccine^30^. Meanwhile, several interrelated barriers to vaccine uptake have been identified for children with zero-dose and those who are under-immunised, including gender, poverty, geographic access, service experience and the non-alignment of the NIP with the needs of vulnerable people^31,32^. These barriers constitute an impasse that reinforces the tendency of caregivers not to immunise their children.

Therefore, this study aims to assess the underlying influences on immunisation decision-making among caregivers of children Under-5, and the factors shaping these behaviours.

## Methods

The study employed a qualitative design in the form of a Focus Group Discussion (FGD). It was conducted in accordance with the guidelines and approval of the Nigeria’s Health Research Ethics Committee (ref. no.: FHREC/2023/01/48/04-03-23). Written informed consent was obtained from all participants. While acknowledging that the researcher’s background, experiences, and beliefs may influence the collection, interpretation, and analysis of data, steps were taken to remain aware of unintentional perspectives, minimise bias, and ensure that the voices of participants are accurately represented. Reflexive practices, including keeping a reflective journal and discussing interpretations with colleagues, were employed throughout the study to enhance the transparency and trustworthiness of the findings.

Sampling design.

The study sample comprised caregivers of children Under-5 who were purposively recruited from four predefined, stratified clusters in the North-west, South-west, South-east and South-south geopolitical regions. Eligible participants were then aggregated in group meetings. The primary target group comprised mothers, fathers or legal guardians of children aged 0–5 years, who are referred to as caregivers in Nigeria. The sample population was further stratified into four sub-clusters (Kano, Osun, Enugu and Edo states) using a simplified cluster sampling approach^33,34^. One community from was then randomly selected from each sub-cluster, from which participants were purposively drawn. The selected communities in the sub-clusters from which participants were drawn had adequate geographical representation and provide immunisation services through healthcare facilities for at least three years prior to the study. At least seven caregivers with children Under-5 were included from each cluster, generating a total of 28 participants.

The participants were representative of the Nigerian population in terms of age, gender, socio-economic status, religion, and region of origin. Each participant received an information sheet detailing the aims and expected outcomes of the study. This was accompanied by an informed consent form, which was signed by all participants. The FGDs were audio recorded and explored the perspectives of caregivers on the behavioural influence of childhood immunisation decision-making.

Themes explored.

The FGDs were guided by a carefully designed semi-structured instrument. It explored themes such as: *knowledge about childhood immunisation* (“What comes to your mind when one talks about childhood immunisation?”); *immunisation demand* (“In your experience, how will you consider current immunisation acceptance?”); *factors driving low immunisation coverage* (“In your view, what could be factors responsible for low immunisation uptake and why?”); *Importance of vaccines to a child’s health* (“How important do you think vaccines are to a child’s health? Follow-up: How important do you think for a child to received none, some or all of these vaccines?”); *masculinity* (“When it is time for children to get vaccinated, the mother would need permission to take the child to the clinic for immunisation? Follow-up: How does the final decision on child’s immunisation rests on the fathers? Why is his permission important? Is it because of culture, fathers are head of the house, fathers better understand immunisation, God/Allah commanded it?”). Others are: g*ender disparity: boys versus girls* (How do you see immunisation between boys and girls…do you think immunisation is more important for girls than boys or vice versa…Why do you think so? ). *Misinformation* (“What do you think about all the information going around about immunisation? Follow-up…some vaccines generally contain chemicals that are harmful…prayers are more effective in preventing diseases…some vaccinations are designed to reduce our population). *Religious beliefs* (“How would you consider religious beliefs and immunisation…does it go well with religious beliefs?”). *Immunisation schedule* (“Nigeria has a schedule of vaccines for children. Do you think people want their children to receive none, some or all childhood immunisation and why?”). *Immunisation intention* (“Has anyone here received Hepatitis B vaccine? If so, why did you take it? If not, would you like to receive vaccination for Hepatitis B?”). *Impact of COVID-19 on immunisation services* (“Has the COVID-19 pandemic affected your views about immunisation generally. If so, why? If not, why?”).

### Data analysis

The data were analysed using a meta-aggregation approach, which summarises data in a stepwise process to develop themes based on predefined concepts^35,36^. After analysing each individual transcript (first-order data), confluent and deviant views based on sorted insights were identified (second-order data) and categorised based on the aggregation of subject outcomes (third-order data). A similar process was used for themes that emerged inductively from the data. All discussions were audio recorded and subsequently transcribed verbatim. The transcribed data were coded according to the participant’s identifiers (RS1 – RS7) and the corresponding to the clusters (f1 – f4), e.g., f1001-7, f2001-7, f3001-7, and f4001-7.

## Results

### Drivers of low immunisation uptake

#### Inadequate knowledge about childhood immunisation

Caregiver’s knowledge of immunisation services was mixed, or at best, inadequate. There was a lack of understanding about which diseases can and/or cannot be prevented by immunisation. Most caregivers believed that all childhood diseases could be prevented by immunisation.

“I think immunisation is an act of making someone resistant to infectious diseases"…f1RS1. “I will say it is a kind of medicine given to a child to prevent any kind of disease"…f2RS1. “…immunisation is just about giving children injections against diseases"…f2RS2.

#### Masculinity (attitudes of fathers/husbands)

**Fig. 1:**
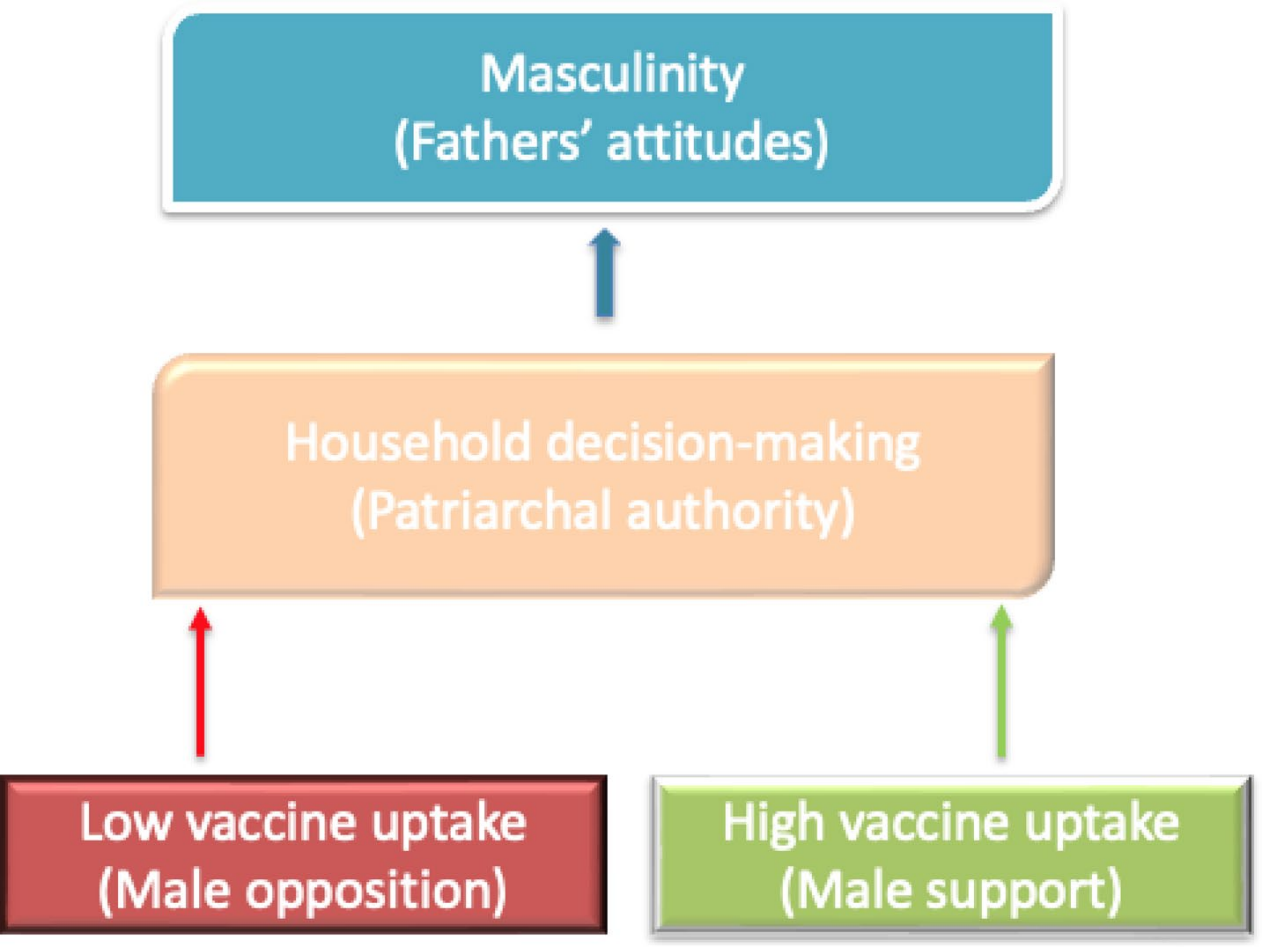
Interaction of masculinity on immunisation uptake.

As shown in Fig. 1, the attitudes of fathers, husbands and other men can either help or hinder the uptake of childhood vaccines. The study found that household decision-making was significantly patriarchal, in the study setting, including on healthcare issues such as childhood immunisation. Thus, the immunisation of children depends on the consent or permission of the father/husband, which is significantly influenced by his attitude towards immunisation. Furthermore, socio-cultural beliefs are crucial in shaping these attitudes.

“We must be carried along"…f2RS4. “…he is the one who takes the major responsibility for the children…his decision is the most important"…f2RS4. “As for me, if anything happens without his consent, it means that I will be held responsible"…f2RS5; " I have to ask permission from him as the head of the family” …f3RS6.

### Gender disparity

**Fig. 2:**
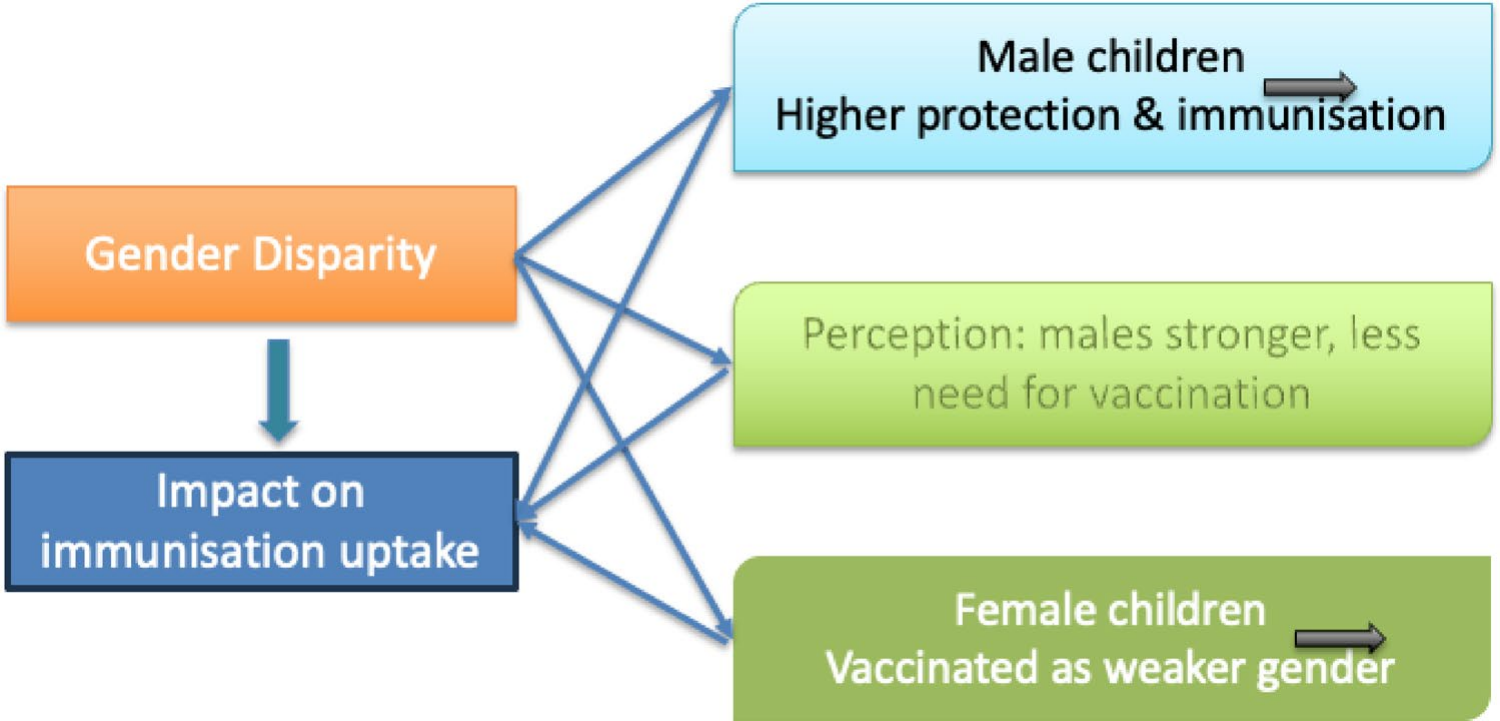
Interaction of gender disparity on immunisation uptake.

Generally speaking, due to various influences, including culture perception of gender roles, boys tend to receive a higher level of protection, including life-saving immunisation, than girls. Similarly, as shown in Fig. 2, the study found that the perception of gender superiority is driven by the knowledge-gap: boys are perceived as having stronger immune systems and therefore requiring little or no immunisation. Consequently, the motivation to immunise girls compared to boys is predominantly driven by the belief that females are the weaker gender and therefore require more protection than stronger males.

“Within the African culture, we put boys first…let’s protect the men more because it is my boy child"…f4RS7; “In the Igbo cultural setting, after you have given birth to six females, he will not even give you the attention to take them for immunisation. But as soon as you give birth to a boy, that day they will immediately ask you if you won’t take that child for immunisation. They will focus on the boy child to protect him from diabolical, physical or other harm"…f3RS6; “Boys are stronger than girls in terms of contracting diseases…they send more of their girls for immunisation than the boys because their bodies are more susceptible to diseases, while boys are stronger due to their immune system"…f2RS7.

### High level of misinformation about vaccines and immunisation

**Fig. 3:**
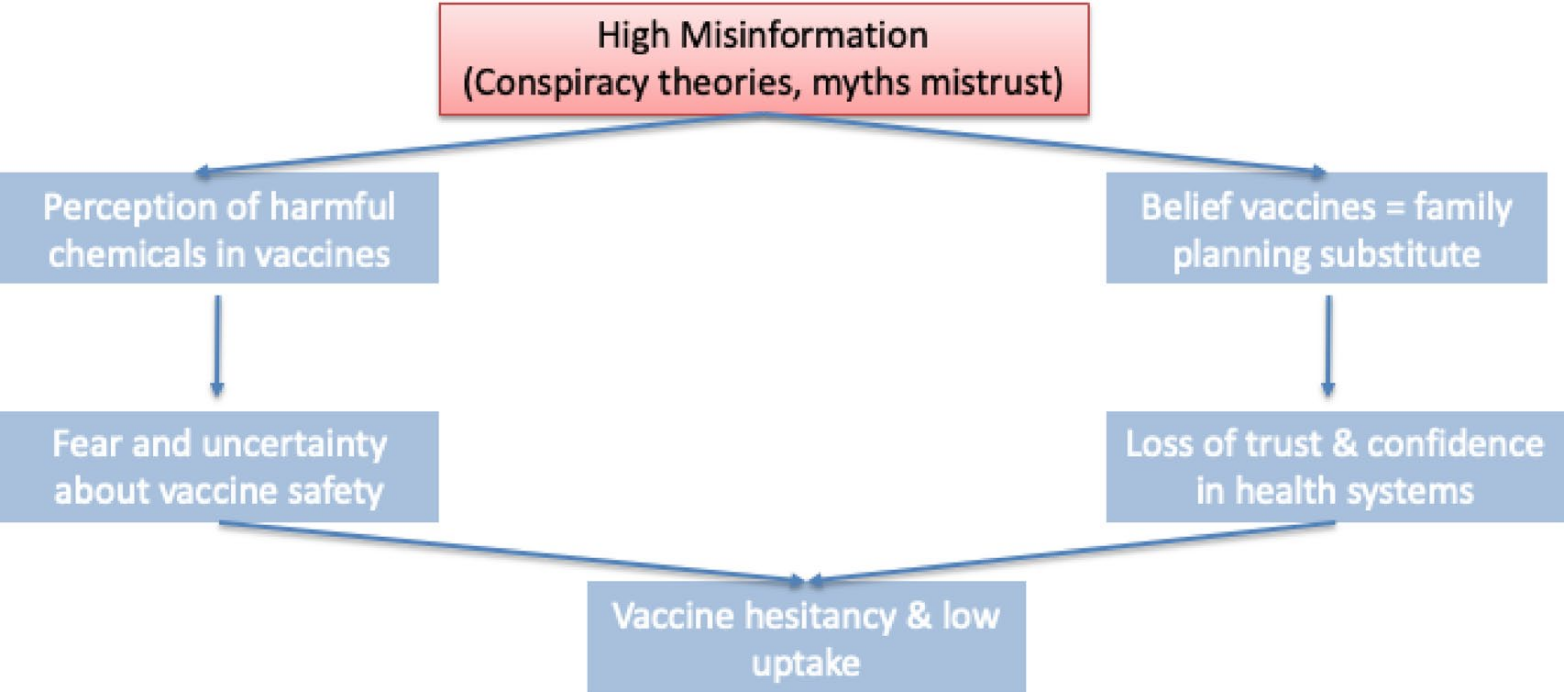
Interaction of misinformation on immunisation uptake.

Misinformation associated with vaccines is high among caregivers, especially conspiracy theories, such as the perception of harmful chemicals in vaccines and are deliberately designed to reduce Africa’s population. As shown in Fig. 3, some people view immunisation as a by-pass for family planning. This has created uncertainties, a loss of confidence and concerns about vaccine safety in general. This behaviour drives vaccine hesitancy and low childhood immunisation uptake in the region.

“I had a relative…it paralysed one of her hands. So, I was actually scared, and my husband was scared too” f4RS6. “I am keen on the issue of the vaccines they are bringing to Africa…people are discouraged because of the fear of female infertility resulting from the vaccines”.f1RS3; “People say it contains chemicals that are harmful to children…somebody said the immunisation in America is different from the immunisation in Nigeria"…f4RS3”.

### Poor immunisation behaviour despite positive health-seeking attitude

Because Hepatitis B vaccination is one of the recommended in Nigeria, using caregivers’ intention to vaccinate their children against Hepatitis B as an indicator of their own vaccination behaviour showed that they had a much stronger interest in vaccines for VPDs, including Hepatitis B. However, despite this positive attitude or intention, there is a low level of Hepatitis B vaccination uptake (a measure of vaccination behaviour) among caregivers; largely driven by inadequate information or knowledge.

“Yes, I would like to receive the Hepatitis B vaccine…but I have not had the opportunity to receive it…f2RS2. “I am very open to it. They brought it to my church, but the crowd was just too much” …f3RS5. “It is very good to take the Hepatitis B vaccine, but in this community, the awareness is very poor. If you ask them what Hepatitis B is, they do not know” …f3RS6.

### Negative impact of COVID-19 pandemic

The COVID-19 pandemic further exacerbated concerns about vaccine safety in general, including routine childhood immunisation. Measures introduced to prevent community transmission, such as lockdowns, as well as the perceived corruption in the handling of the pandemic, misinformation about COVID-19 itself, fear of contracting the virus during hospital visits, and heightened debates about the COVID-19 vaccine affected other healthcare services, including the childhood immunisation service.

“COVID-19 has really affected my attitude towards taking any vaccine. From that time untill now, whenever I go to the hospital, they tell me to take any vaccine, no"…f2RS3. “The COVID-19 vaccine caused so many problems, because of the misconceptions surrounding COVID-19, it made some homes not to take their children for immunisation anymore"…f3RS6.

### Other factors driving low childhood immunisation demand

1. Cost of immunisation: Exploitation of caregivers

Although routine childhood immunisation is free, the study found that caregivers are often exploited by healthcare workers for services at most healthcare facilities.

“The hospitals charge us for immunisation, while the government says immunisation is free…so parents who cannot afford it will not immunise their child"…f1RS1. “The money that they asked me to pay was one of the drawbacks, even though I was willing to vaccinate my child"…f1RS 6. “In my experience, you pay for your own vaccines. Perhaps this action is not from the government, but the healthcare workers just want to extort money for themselves"…f3RS7.

(2) Non-availability or stock-outs of vaccines

The non-availability or stock-outs of vaccines, particularly on immunisation days, contributed significantly to incomplete immunisation and the inherently complacent immunisation behaviour associated with vaccine hesitancy. This was evident in some healthcare facilities.

“Sometimes when you go to the clinic, they don’t have vaccines"…f1RS1. “There are so many health centres that do not offer immunisation"…f1RS4. “My child collects some, but they don’t have them all.” …f3RS1.

(3) Ineffective communication about immunisation schedule and reminder system

Inability to communicate immunisation information effectively, especially regarding the schedule, results in a lack of synchronisation between immunisation providers and communities/caregivers. This negatively impacts on caregivers’ engagement. Some caregivers also fail to immunise their children because they forget, and there is no reminder system in place to assist them. This is particularly prevalent during festivals and among frequent travellers or nomadic families.

“The way someone communicates something to you determines how far you’ll go with that information.” …f4RS2; “I forgot because there was no one to remind me…it was December, and we were preparing to travel home"…f2RS7.

(4) Religious influence

Religious beliefs were a counterproductive factor in vaccine uptake, as certain faith-based teachings and community norms discouraged caregivers from vaccinating their children, resulting in delays or refusal of immunisation. This influence is usually particularly strong when religious leaders openly express scepticism or disapproval of vaccines.

“One of the reasons is religion, especially denominations within Christianity” …f3RS3. “I didn’t see Jesus Christ being vaccinated, nor did I see immunisation in the Bible” …f4RS7.

(5) Poor attitude of healthcare workers

A significant number of healthcare workers exhibit unprofessional behaviour towards caregivers. All too often, they are unempathic, rude and exploitative, which discourages caregivers from visiting immunisation centres.

“Another thing that discourages parents from going to vaccinate their children is the attitude of the healthcare workers. Some of them are just so bad to us” …f3RS6.

(6) Lack of relationship and trust between healthcare workers and the communities

Where there is no trust between healthcare workers and local communities, uptake of healthcare services, including immunisation, is negatively affected.

“The lack of a relationship between the healthcare workers and the communities…trust is key"…f3RS5; “Who and where the healthcare worker comes from matters” …f4RS6.

(7) Fear of Adverse Events from Immunisation (AEFI)

Experiencing an adverse event following immunisation is a significant factor in uptake, especially when combined with a lack of prior awareness of the subject. Health scares caused by AEFI makes caregivers reluctant to expose their children to further immunisations.

“Another thing is the fear of AEFI; it puts them off taking it"…f3RS2; “…because after they give the children the vaccine, some children, after a few days, they will become thin and cry all night, some for days” …f4RS3.

(8) Insecurity

There is a relationship between general insecurity and low immunisation coverage, which is often due to the experience or fear of harm. This has hindered immunisation coverage, particularly in hard-to-reach communities served by mobile clinics and vaccinators. It prevents households from allowing mobile vaccinators, who go door-to-door to immunise this group and nomadic communities, to enter.

“Just like my brother said about insecurity, these days, not everyone who knocks on your gates is someone you want to open the gates for"…f2RS5; “I think the challenge is insecurity in most areas, both urban and rural…you see children being raped, and as a result, people feel that hospitals are not safe either"…fRS6.

## Discussion

**Fig. 4:**
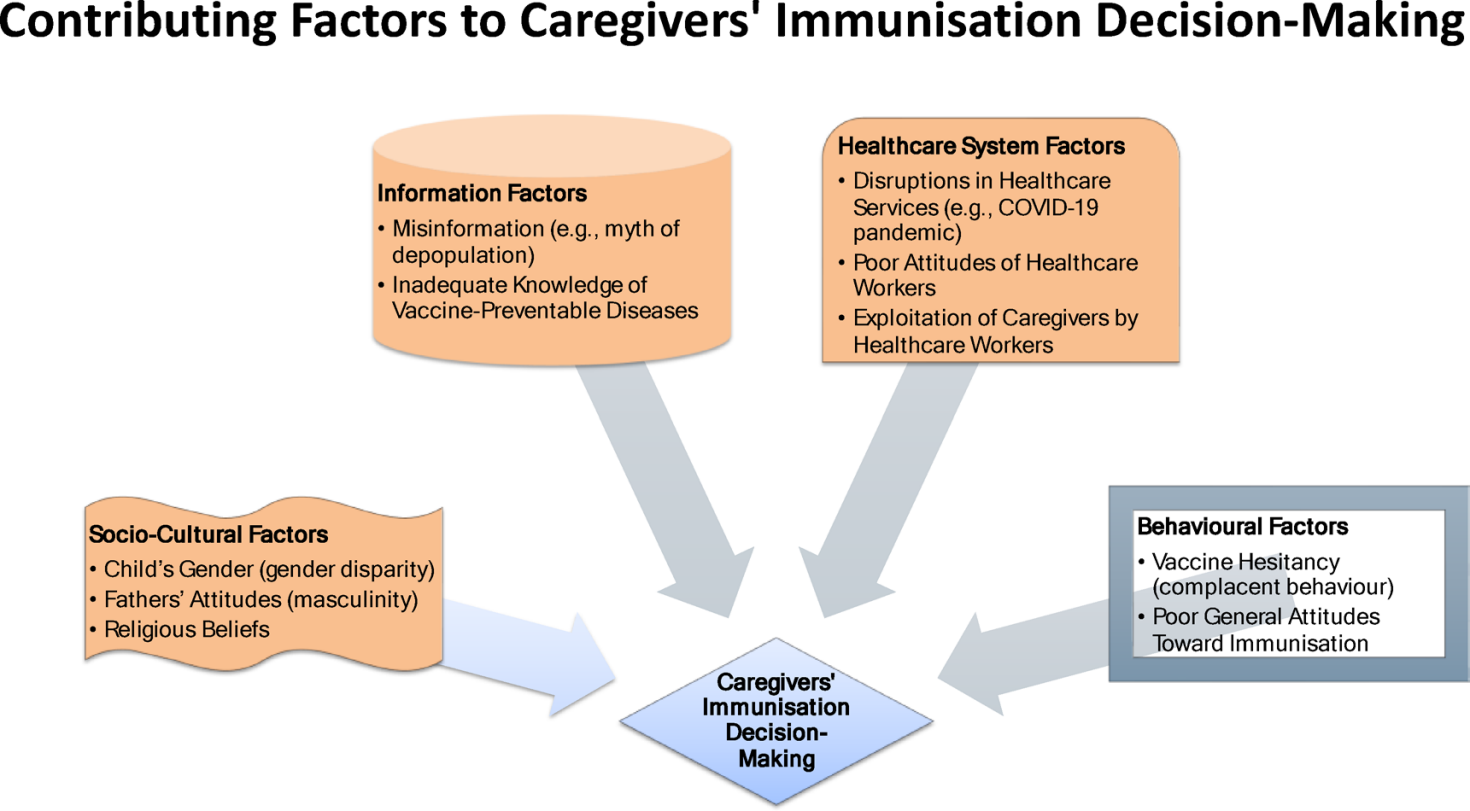
Contributing factors to caregivers’ immunisation decision-making.

The primary aim of this study was to assess the factors influencing caregivers’ immunisation decision-making for Under-5, and to understand the reasons that shape these behaviours. As Fig. 4 shows, the study findings revealed that caregivers’ immunisation decision-making are mainly motivated by: inadequate knowledge about childhood immunisation, especially with regard to vaccine-preventable and non-vaccine-preventable diseases; masculinity (attitudes of fathers/men can hinder or facilitate childhood immunisation); the child’s gender (gender disparity, i.e., the weaker versus the stronger sex); high levels of misinformation or disinformation about immunisation, especially the perception that vaccines contain harmful chemicals designed to sterilise the population (i.e., family planning via backdoor methods); poor immunisation uptake despite positive health-seeking attitudes; and the spillover effects of the COVID-19 pandemic, which further exacerbate concerns about vaccine safety. Other factors include the exploitation of caregivers by healthcare workers, vaccines unavailability or frequent stock-outs, leading to complacent behaviour associated with vaccine hesitancy; ineffective communication of the immunisation schedule and poor reminder systems; religious beliefs; poor attitudes of healthcare workers; a lack of trust between healthcare workers and the host communities; and fear of AEFI and general growing insecurity.

### Closing the immunisation knowledge-gap

Childhood immunisation appears to be a priority for caregivers, as the study found an increased sense of protection by caregivers for their children’s health, due to its prophylactic capacity, hence the importance attached to immunisation despite the cost (financial burden), AEFI, and other barriers. However, inadequate knowledge of immunisation continues to hinder uptake in limited-resource settings, particularly in Nigeria. Previous studies have shown that this impairs caregivers’ ability to distinguish between vaccine-preventable and non-vaccine-preventable diseases^12,19^. Unfortunately, this study shows that this phenomenon continues to affect immunisation coverage in the country post COVID-19 pandemic. This knowledge deficiency breeds a lack of confidence and trust, consequently leading to complacent behaviour among caregivers.

If children continue to suffer or die from diseases despite being immunised, caregivers’ confidence in vaccines is likely to diminish. This is because the knowledge available to them suggests that once a child has been vaccinated, they are protected from all diseases or death. This misconception is a recipe for vaccine hesitancy. Also, it is more likely to lead to complacent behaviour based on the perception that immunisation is just a lie, ineffective or after all unimportant because it does not prevent all childhood diseases. “In my own environment, I see that some people still don’t accept it. They don’t consider it important"…f3RS5. The perception among caregivers that immunisation provides blanket insurance or protection against disease (vaccine-preventable or not) or death is misleading and needs to be corrected urgently.

Therefore, it is crucial to educate the general population with adequate knowledge about vaccines and immunisation. Community health education interventions are needed to educate caregivers and the general population about diseases that can be prevented by vaccines and those that cannot. Similarly, the retraining of healthcare workers, who act as frontline health educators at the community healthcare facilities, is paramount to conveying accurate vaccines efficacy. Individuals and groups who thrive on misinformation, disinformation or conspiracy theories about immunisation can easily exploit such knowledge-gaps to sow seeds of mistrust about immunisation.

Given that the study found that a significant proportion of the population still lacks critical information about immunisation, especially in rural areas, issues related to inadequate information or low levels of awareness lead to misconceptions about the purpose of vaccines. This affect caregivers’ engagement and inevitably impacts vaccine uptake. “A lot of people are not aware…in rural areas; they don’t even know they are supposed to take these vaccines” .f1RS3. For immunisation campaigns to be effective, information should be disseminated through multiple media sources, especially visual communication^18^. Campaigns should provide detailed information about what vaccines are, what they do, what they cannot do, and where to obtain them. “Create more awareness just to tell people about it and the right place to get al.l these things.I think that will help lots of people to develop the confidence to begin to take them”.f2RS2.

### Impact of vaccine misinformation or rumours

In the study setting, misinformation appears to have a dominant effect on caregivers’ immunisation behaviour. There is a high level of misinformation about vaccines being harmful or containing harmful chemicals, as well as inaccurate information and communication about AEFI in children. This has a negative impact on their attitudes towards immunisation, particularly in rural areas. “I was told that if I get vaccinated and experience pain in my arm, I might have to have it amputated"…f1RS3. “So many people, you see their leg. They say it is the result of the immunisation they took” …f4RS4.

Misinformation attributed to AEFI is associated with unexplained or poor communication of the expected or likely adverse events that predispose the circumstances, conditions and events surrounding childhood immunisation. Knowledge of AEFI should be a critical component of ANC to ensure that caregivers receive adequate education about AEFI. This activity must also extend to Traditional Birth Attendants, who do not interface with healthcare facilities, to avoid a gap in the community healthcare continuum. When information about adverse events is available, prior knowledge of the symptoms of AEFI and how to respond to them can help to avoid the gaps filled by misinformation about AEFI, such as those expressed by the participants above.

Also, there was a high level of misinformation associated with the vaccines themselves was evident among caregivers (both fathers and mothers), especially conspiracy theories such as the idea that vaccines contain harmful chemicals intended for population control or that may compromise the future reproductive health of children. Vaccine-related misinformation creates uncertainties and fears about vaccine safety in general, which in turn affects attitudes towards immunisation. The study shows that a key driver of vaccine misinformation is ineffective communication about vaccines, which leaves room for conspiracies and rumours to thrive.

Rumours generally thrive where the appropriate medium or media to disseminate the correct information is absent or dysfunctional. Before people are inundated with fake news about vaccines, it is imperative to provide them with the correct information to encourage positive vaccine behaviours, particularly when caregivers are still pregnant (i.e., during ANC). The lack of effective strategies for communicating information about vaccines and immunisation to caregivers, especially using health informatics, remains a sore point in the functioning of health systems in most developing countries, including Nigeria.

One of the factors driving the depopulation perception or misinformation is the low uptake of family planning services, coupled with the aggressive promotion of this healthcare intervention in the SSA region, particularly in Nigeria. There is a belief that family planning is un-African as a public health intervention and was designed to target population growth in the region^37^. This belief is widely held by religious and traditional leaders^12,28^. Thus, immunisation as a public health service has become an unintended victim of this push-pull conundrum in the family planning debate. Some people perceive immunisation as a way of indirectly sterilising children, thereby reducing their future fertility^12,19,20,28^.

### Debunking misinformation and implications for practice

The mythical theory of de-population has caused significant setbacks to various healthcare programmes in Africa, particularly immunisation programmes^3,38^. Similarly, several attempts and interventions have been made to debunk the theory and re-educate the population, but with limited success, as equally observed in this study^39,40^. It is noteworthy, however, that these debunking methods may not have been grounded on any evidence-based theories, which explains why current interventions to address misinformation have been ineffective.

This study found the “truth sandwich” theory, developed by the Robert Koch Institute (RKI) to debunk common misinformation about immunisation, to be a critical asset or valuable tool^41,42^. Although the truth sandwich model was designed for patient consultation scenarios, it is also well suited to interventions targeting misinformation in limited-resource settings, where trust in healthcare workers as a reliable source of information about immunisation has been shown to be very high^13,14,19,20^.

First, the *fact or truth* about immunisation must be stated, including the fact that, although they do not offer 100% guarantee, vaccines are safe and effective against diseases that can be prevented by immunisation. If such a scenario exists, interventions must simplify the facts about immunisation in a way that aligns with established knowledge about the effectiveness of a particular, non-controversial vaccine. Secondly, *myth*,* conspiracy or misinformation* about immunisation must be identified and echoed. In this case, as this study has identified, the narrative that childhood vaccines contain family planning medications or therapies designed to impair the reproductive capacity of the African population must be mentioned as a familiar narrative. Thirdly, the *fallacies* should be explained, particularly how and why the misinformation is not only inaccurate and misleading, but the origin and/or possible motives should also be explained if known to be factual. Finally, the interventions targeting misinformation must repeatedly reinforce the *facts* until they provide an alternative causal explanation that remains the only information taken away.

**Fig. 5:**
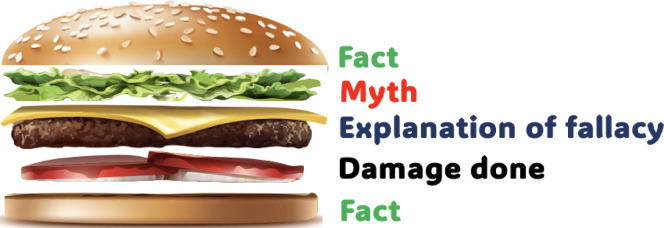
The sandwich model for tackling vaccine misinformation.

The truth sandwich model could be improved by explaining the *damage done* by the myth or the consequences of the myth (e.g., that low vaccine uptake makes populations vulnerable to disease outbreaks). I.e., practical examples should be used to illustrate the real-world consequences of the damage caused by vaccine myths and misinformation. Myths and misinformation are major ingredients that fuel vaccine hesitancy, leaving populations vulnerable to disease outbreaks and preventable deaths, as well as putting increased strain on the healthcare systems, especially in limited-resource settings. As shown in Fig. 5, explaining the *“damage done*” should come before the final layer, i.e., reinforcing the “*fact”* about vaccine safety and efficacy.

### Gender as a driver of demand for immunisation

In this study setting, gender plays a significant role in immunisation decision-making and behaviour, albeit for different reasons. A dual approach to immunisation appears to exist, which can explain the disparity in coverage observed between boys and girls in this study setting and across SSA more broadly. This is dangerous for vaccine uptake and also a recipe for vaccine hesitancy and outbreaks of VPDs.

Although both male and female children have an equal need for immunisation, uptake between boys and girls, a disparity accentuated by cultural beliefs and perhaps misinformation. Given the recent evidence suggesting this^12,19^, it was not surprising to find that a child’s gender influences immunisation uptake in Nigeria. However, the finding that a child’s gender can influence immunisation uptake in both directions is novel and a unique outcome of this study. That is, being a boy or a girl can both influence immunisation behaviour. While it is not entirely unexpected that male children receive more protection, including immunisation, than female children, this is partly due to the influence of culture. However, it is surprising to find a perception of male superiority, given that males are generally considered to have stronger immune systems and therefore require less or no immunisation. Conversely, the motivation to immunise girls compared to boys is driven by the notion that girls are the weaker sex and therefore require more protection than supposedly stronger boys.

The centrepiece on immunisation, which is dominated by a preference for boys over girls, was found to be primarily due to the guardrail on the economic and financial provider of the households in the community. A man needs to be strong and healthy, so if immunisation can guarantee this, then he becomes the priority. The idea of immunisation as a form of prophylaxis or harm prevention for the households meant that the egghead of the family had to be at the centre of protection to ensure the household’s economic survival.

The gender disparities in immunisation found in this study are significantly influenced by cultural and socio-economic considerations, as well as misinformation and inadequate knowledge, which in most cases are not peculiar to immunisation. Therefore, cultural influences and misinformation appear to be the most prominent drivers of gender differences in childhood immunisation uptake. This study serves as a reminder that gender inequity remains prevalent in key areas of the SSA and has historically affected women disproportionately from social, economic and political perspectives. Nevertheless, efforts to close this gap have come a long way, and it is much narrower than it used to be. However, this study reminds us that much more needs to be done to close this gap, especially within the healthcare system. Over the past few decades, societies, policies and populations have evolved to overcome gender discrimination against women, and it was known that this has also affected immunisation, particularly among children Under-5.

Finally, the study’s novelty outcome can be positioned in many ways, but most importantly as follows: The d*ual directionality of gender as a driver of immunization uptake.* I.e., previous studies often emphasise a one-sided gender problem, typically that girls receive fewer immunisation due to cultural devaluation. However, this study shows that both boys and girls may be disadvantaged in terms of immunisation uptake for different reasons, as highlighted above. This paradox has not always been documented, so this study introduces a new dimension to the gender – health debate. Secondly, *the interplay between masculinity and household economics*. I.e., the notion that immunisation is linked to protection of the household’s economic provider (i.e., future men) shows the rare intersection of gender norms, economic rationality and masculinity. This means that immunisation behaviour is directly linked to household survival rationality and patriarchal norms in a way that has not been clearly articulated before. Third, the intersection of c*ultural and misinformation.* I.e., although immunization uptake is known to be influenced by misinformation, but this study shows how misinformation and cultural beliefs reinforce gendered perceptions (e.g., that boys are stronger and need fewer vaccines). This deepens the understanding of how misinformation not only distort facts about immunisation but also become intertwined with gendered worldviews. Lastly, *the contextual nuance beyond the study setting.* By demonstrating that these dynamics vary across different geographical settings, the study shows that gendered immunisation behaviours are not monolithic but are shaped by local socio-cultural contexts.

### Masculinity

Fathers can make or mar immunisation uptake. Their individual perceptions and attitudes towards immunisation have a significant impact on whether children within households are immunised. More importantly, other factors also that influence these attitudes include the costs involved (men are perceived as being responsible for these costs), the mother’s fear of being blamed for any adverse event or incidence (s) in the child that may be directly or indirectly related to immunisation, and the importance of respecting men’s position as heads of households. Immunisation without the father’s consent or permission can have socially undesirable consequences. Although mothers are more likely to carry out the practical aspects of childhood immunisation, the decision to vaccinate or not depends significantly on how fathers feel or think about vaccines or immunisation. Therefore, their attitudes play a key role in this decision and influence whether children start, continue or complete the full course of childhood immunisations. One caregiver said: “He would be the one to provide transport and other fees that I am going to use. So, I will take permission from him because of the money I would collect and other things"…f3RS1.

For socially desirable reasons, some men avoided discussing the topic during this study. Nevertheless, the majority of male participants agreed with the notion that they best understand their households best and should therefore make decisions about them. Some male participants, for socially desirable reasons or to appear politically correct, did not explicitly agree with the former’s deposition. Thus, on the one hand, they gave answers that suggested immunisation decisions are mutual between mothers and fathers, and that permission is unnecessary since no harm is intended by immunising the child. However, these socially desirable responses were accompanied by a caveat that qualified them, thus further confirming the assertions of the former. For example, one of them alluded to the following: “It is not necessary, however, anywhere my wife goes, she would have to have my consent"…f2RS6.

The study shows that when fathers’ knowledge and confidence in childhood immunisation is high, mother’s attitudes towards immunisation are generally positive, regardless of their own disposition. However, the reverse is not true. In other words, negative attitudes towards immunisation among fathers affect mothers’ ability to immunise, regardless of the mother’s own attitudes. Therefore, fathers should support mothers by showing an interest in their awareness of childhood immunisation and getting personally involved in ANC and postnatal care activities. It is evident therefore, that a lack of knowledge about the importance of vaccines and negative perceptions among fathers can have serious implications for mothers when it comes to childhood immunisation. In addition, the cultural environment surrounding routine immunisation is strongly male-dominated (patriarchal), with men as the head of households and women expected to submit to their authority. Decision-making within households in the study setting demonstrates the complex cultural influences on behaviour regarding immunisation, which are dominated by masculinity. “The man is the head of the household. He owes his wife protection; his wife owes her husband obedience”^43^.

Inadequate knowledge and misconceptions about vaccines and immunisation among fathers could discourage mothers from seeking immunisation services. Also, if fathers have negative perceptions of immunisation, the decision to immunise children could become collateral damage in the event of disagreements within the household. “Sometimes husbands and wives disagree, the husband would think that he is the head, and he decides a lot about what the family should do…he might prevent his wife from taking their child for immunisation.“…f2RS3. Masculinity is therefore clearly a threat or barrier to immunisation uptake, especially in the study setting. Interventions that specifically target men or fathers therefore need to be promoted and scaled up. In this environment, children whose fathers are hesitant to have them vaccinated are less likely to start, continue or complete the full immunisation course.

### The COVID-19 pandemic factor

The negative impact of the COVID-19 pandemic on childhood immunisation will take a long time to reverse because immunisation is one of the healthcare services affected by the pandemic^29^. The pandemic exacerbated existing stressors and barriers, setting back progress in immunisation coverage by many years and resetting norms about attitudes towards vaccines. A key finding of this study is that the country’s healthcare system must develop robust operational guidelines and protocols for pandemic preparedness. This would prevent some of the draconian, non-evidence-based intervention measures taken during the pandemic and which further complicated and almost caused the collapse of the healthcare system in Nigeria. The SSA region experienced a double tragedy: the pandemic itself and the negative impact of the response to it, which further crippled the lives and livelihoods of most communities. These scenarios, coupled with the population’s backlash, created fertile ground for the spread of misinformation not only about the pandemic itself, but also about everything directly or indirectly related to it, such as healthcare services and vaccines in general.

### Exploitation of caregivers

The exploitation of caregivers in healthcare facilities requires special attention. Previous studies have identified similar behaviour, yet it persists^12,19^. This has been identified as a major barrier affecting access to immunisation, creating psychological constraints that hinders uptake, particularly in limited-resource settings^12,19^. While caregivers may be willing to immunise their children, affordability constraints may be the greatest barrier, particularly in limited-resource settings, such as the SSA region. As these participants further posited:

“The money that they asked me to pay was one of the determinants…despite the fact that I am willing to vaccinate my child"…f1RS 6. “Let government make immunisation services to be free"…f2RS1. “If I carry my child for immunisation and I don’t have the money I might not be able to get it. Let them help us and make immunisation free"…f2RS4.

Therefore, addressing issues such as illegal fees, charges and exploitation by healthcare workers could minimise the turn-off of willing caregivers.

### Training and retraining of healthcare workers

The success of immunisation programmes depends critically on healthcare workers. Therefore, considerable attention should be paid to training and retraining of personnel involved in these programmes. In addition to the signing of codes of conduct, retraining and establishing best practice guidelines and protocols in healthcare facilities, efforts should address the incessant poor attitudes and exploitative tendencies of some healthcare workers, as observed in this study. Immunisation demand cannot thrive where systemic unprofessional behaviour by healthcare workers towards caregivers is prevalent, as this is evidence of a fractured healthcare system, lacking leadership and systemic guidelines. Healthcare workers are not only exploitative, but also unsympathetic and often rude to caregivers. This behaviour is systemic, i.e., not limited to immunisation services, and therefore requires drastic overhaul, especially in rural areas.

### Role of incentives

Incentivising healthcare services has been shown to be successful, particularly in settings with limited resources^44–46^. Such motivation is needed in this setting, where poor attitudes towards healthcare, among other barriers, have limited immunisation uptake despite positive health-seeking intentions among caregivers. Incentivising caregivers would boost participation in immunisation programmes and force the healthcare system to rethink its approach: “.support parents with things like mosquito nets”.f2RS2. “.support parents even if it’s just a mosquito net, because the nurses have discouraged many mothers”.f4RS2.

#### Reminder system

Immunisation campaigns without an effective reminder system will not adequately address the phenomenon of high dropout rate, incomplete immunisation and zero-doses in Nigeria. Therefore, redesigning or leveraging various new communication tools to enhance caregiver follow-up and reminders is critical. Phone calls, text messages and other reminders are essential to help caregivers not only start but complete the routine immunisations. “I think there should be sending of messages to remind parents…” RS2; “They should call parents” …RS3.

### Heightened insecurity and immunisation demand

Negative attitudes towards immunisation are increasingly being driven by the relationship between higher levels of insecurity, a lack of trust and a fear for one’s safety, and low vaccine uptake. Evidence shows that in settings where violence and victimisation are prevalent or widespread, child health and immunisation uptake suffer in particular^17,47,48^. This is because children of victimised caregivers are less likely to be fully immunised and are more likely to avoid visiting public healthcare facilities for care^47,48^. Therefore, addressing the issue of safety and caregivers’ fear or mistrust of the healthcare system/workers is key to encouraging positive healthcare-seeking behaviour and increasing immunisation uptake.

This study has limitations that should be acknowledged. Although the qualitative research approach allows for in-depth exploration of caregivers’ experiences and reveals rich contextual details, the data collected may be insufficient to generalise the experiences of everyone in the setting, or to address the heterogeneity of experiences across communities. Sampling constraints were also noticeable, as the study could not include all six geopolitical zones of the country as study clusters; rather, only four were included. Furthermore, the use of FGDs may have inadvertently introduced a social desirability bias, which could jeopardise the heterogeneity of caregivers’ views. However, despite these limitations, the cross-sectional nature of the study, realignment of the clusters to fit national representation, use of open-ended questions, prioritisation of participant anonymity to foster openness, saturation of qualitative data and depth of insight are sufficient to support the reported conclusions.

## Conclusion

This study established that the child’s gender, fathers’ attitudes, weaponised misinformation and the impact of COVID-19 pandemic, among others, were fundamental factors influencing caregivers’ decision-making about immunisation in limited-resource settings such as Nigeria. Due to these links with immunisation behaviour, the likelihood of boys having higher immunisation coverage than girls, or girls having higher immunisation coverage than boys, is embedded in a culture of masculinity reinforced by patriarchy.

Therefore, interventions must first address the root causes of these linkages, as well as the social norms and cultural practices that enable them. Societies with high levels of masculinity are more likely to have lower immunisation coverage among boys when beliefs about immunity are gendered, and lower coverage among girls when beliefs about the weaker sex are prevalent. Nevertheless, cultural practices and norms in Nigeria that are based on protection via immunisation puts female children at a disadvantage, as they are less likely to be immunised than male children. New ways of communicating immunisation information to caregivers are needed, including schedules and reminders, and these must be integrated into the national framework. Furthermore, the sandwich model for addressing vaccine misinformation (Fig. 5) provides a new approach to tackling myths and misinformation about vaccines. Other recommendations include using gender-sensitive or gender-transformative intervention approaches to address patriarchal norms that prioritise male domination in households’ health decision-making; using male peer role models to champion supportive health-seeking behaviours; integrating traditional and religious leaders into immunisation programmes to help shift norms around gender and immunisation; and retraining healthcare workers to use culturally relevant narratives to debunk beliefs such as “boys are stronger and need fewer vaccines; girls are weaker and need more vaccines”.

Further research is recommended, particularly quantitative research, to understand the causal relationship between the outcomes of this study and vaccination intention, behaviour or decision-making of caregivers. This would further help explain how changes in one factor affect caregivers’ vaccination decision-making for children Under-5, facilitating interventions that could improve immunisation coverage.

## Data Availability

The datasets used and/or analysed during the study are available at https://osf.io/537pt/. Further data can be requested from the author through [gbadebo.adeyanju@uni-erfurt.de](mailto: gbadebo.adeyanju@uni-erfurt.de) .
